# Characteristic of Przewalski horses population from Askania-Nova reserve based on genetic markers

**DOI:** 10.1007/s11033-023-08581-4

**Published:** 2023-06-26

**Authors:** Adrianna D. Musiał, Katarzyna Ropka-Molik, Monika Stefaniuk-Szmukier, Grzegorz Myćka, Agnieszka Bieniek, Nataliya Yasynetska

**Affiliations:** 1 Department of Animal Molecular Biology, National Research Institute of Animal Production, 32-083 Balice, Poland; 2 Biosphere Reserve Askania-Nova, Kherson region, 75230 Ukraine

**Keywords:** *Equus przewalskii*, mtDNA, Chromosome Y, *TBX3*, *MC1R*, Molecular markers

## Abstract

Przewalski horses are considered the last living population of wild horses, however, they are secondarily feral offspring of herds domesticated ~ 5000 years ago by the Botai culture. After Przewalski horses were almost extinct at the beginning of the twentieth century, their population is about 2500 individuals worldwide, with one of the largest breeding centers in Askania-Nova Biosphere Reserve (Ukraine). The research aimed to establish the maternal variation of Przewalski horses population maintained in Askania-Nova Reserve based on mitochondrial DNA hypervariable 1 and hypervariable 2 regions profiling, as well as, analysis of Y chromosome single nucleotide polymorphism unique for Przewalski horses, and coat color markers: *MC1R* and *TBX3*. The mtDNA hypervariable regions analysis in 23 Przewalski horses allowed assigning them to three distinctly different haplotypes, showing the greatest similarity to the *Equus caballus* reference, the *Equus przewalskii* reference, and to extinct species—*Haringtonhippus*. The Y chromosome analysis using fluorescently labelled assays differentiated horses in terms of polymorphism (g731821T>C) characteristic of *Equus przewalskii*. All male individuals presented genotype C characteristics for Przewalski horses. The polymorphisms within the coat color genes indicated only native, wild genotypes. The Y chromosome and coat color analysis denied admixtures of the tested horses with other Equidae.

## Introduction

Przewalski’s horse genome falls outside a domestic horse’s group, approximately 38,000–72,000 year BP [1]. Przewalski horses are considered the last living population of wild horses [2], however, the previous research based on whole genome sequencing indicated that they are feral descendants of the horses domesticated about 5000 years ago by the Botai culture in present-day Kazakhstan [3]. The species’ history remains unknown for nearly five millennia until Przewalski horses were rediscovered as a free-range population deemed to be wild about 150 years ago [4]. In the twentieth century, Przewalski horses population went through several bottlenecks and almost became extinct but survived from a captive herd as a result of enormous conservation and reintroduction efforts. After a drastic decrease in individual numbers, and based on pedigree data, 11 pure species founders are distinguished including three Old Askania-Nova line founders, one New Askania-Nova line founder, three Old Prague line founders, and four Munich (US) line founders [5]. However, the population cannot be genetically pure, as in the early 1900s, several zoos interbred their Przewalski horses with domestic horses to save the species and started to reproduce another two founders: a domestic horse and domestic/Przewalski hybrid [4, 6]. Now, their population consists of no more than 2500 individuals worldwide including 900 horses in Europe and 1360 in Asia [7].

One of the largest breeding centers for Przewalski horses is Askania-Nova Biosphere Reserve (Ukraine). The first 10 Przewalski horses were imported to Askania-Nova from Mongolia in 1899–1903 and by 1945, 37 live foals were born but a decline in interest in horses, inadequate breeding conditions, extremely difficult exchange of producers and, as a result, breeding in strict inbreeding caused the degeneration of the entire population and almost extinction in the 1940s. Purposeful breeding work on this species, which led to its preservation, began after the publication in 1959 of the first Przewalski horse studbook [8]. Nowadays, the population in Askania-Nova is maintained at about 60–70 individuals which are kept under semi-free conditions in large enclosures with natural steppe vegetation as well as in small zoo exhibition paddocks without grass [9].

Previous studies on mitochondrial DNA (mtDNA) identified three unique haplotypes in Przewalski horses, none of which were found in modern horses—two similar ones and one substantially divergent from them [10]. However, recent studies indicate evidence of four Finnhorse and one Latvian horse individuals carrying mitochondrial haplogroup previously confined only to Przewalski horse [11].

The basic coat colors genetic control in horses resides at two genetic *loci*, namely Extension (E) and Agouti (A) which are responsible for the three basic colors occurrence—black, bay, and chestnut [12]. The most common color seen on Przewalski horses is bay with Dun dilution. It has been shown that Przewalski horses, as well as the Siberian horses of that time, do not have the black pigmentation recessive alleles ‘a’ in *ASIP* gene, which is possibly related to their living in the Asian steppe’s adaptation [13, 14]. The archaeological samples studies indicated that during domestication the selection pressure had a great impact on coat color variations [13]. It was hypothesized that ancestral horses were characterized by black-based patterns [12], and all Siberian and European Pleistocene horses were bay or bay-dun [13]. The ‘fox’ color carriers caused by *MC1R* recessive alleles ‘ee’ should not be present in Przewalski horses, however, the previous research showed the ‘fox’ color alleles presence in 15% of the tested Przewalski horses [14]. The results may be the consequence of the population crossbreeding in the early days of the conservation efforts. In *TBX3* gene for Dun-related trait; there is only one characteristic genotype observed in all Przewalski horses. Horses with this genotype show a diluted body color and have dark points called primitive markings including dorsal stripe and leg barring [15].

The research purpose was to establish the maternal variation of the Przewalski horses population maintained in Askania-Nova Reserve based on whole mtDNA hypervariable regions profiling, as well as, analysis of Y chromosome SNP unique for Przewalski horses, and coat color markers: *MC1R* (‘fox’) and *TBX3* (Dun).

## Materials and methods

The analyses were carried out on hair follicle samples derived from 23 Przewalski horses population. The analysed individuals belonged to three Przewalski horses lines (Prague, Munich, and New Askania-Nova) living in the Askania-Nova reserve (Ukraine). The tested horses (13 stallions and 10 mares) represented all the lines of the Askania-Nova reserve population. The 21 animals were sampled after death and the other two when disturbed according to the classic procedure for zoos including branding animals and assigning them an international breeding number. During the veterinarian procedure, the hair follicle samples were taken from the mane or tail of the horses.

The DNA was isolated using Sherlock AX kit (A&A Biotechnology) according to the manufacturer’s protocol and stored at −20 °C. For the mitochondrial DNA hypervariable regions amplification, three PCR products were designed covering a total region of 1062 bp (X79547.1; Table 1). Amplification was performed on all 23 individuals using the Phanta Ready Mix (Vazyme Biotech). PCR products were cleaned with the enzymatic method using the EPPiC Fast reagent (A&A Biotechnology) and used as a template for sequencing by the Sanger method. The PCR for the sequencing reaction was performed for each amplicon (69 samples in total) with the BigDye Terminator v3.1 Cycle Sequencing Kit (Thermo Fisher Scientific) and the products were repurified with the BigDye XTerminator Purification Kit (Thermo Fisher Scientific). The capillary electrophoresis was performed on 3500xL Genetic Analyzer (Thermo Fisher Scientific) using POP-7 ™ Polymer for 3500/3500xL (Thermo Fisher Scientific) and the results were analysed using FinchTV (Geospiza, Inc.), BLAST and Variant Analysis (Thermo Fisher Cloud) as well as compared with the GenBank reference sequences belonging to the species: *Equus caballus* (X79547.1), *Equus asinus* (MK896302.1), *Equus asinus somalicus* (MG885769.1), *Haringtonhippus francisci* (KT168329.2), *Equus grevyi* (NC020432.2), *Equus kiang* (NC020433.1), *Equus hemionus* (NC018782.1), and *Equus przewalskii* (KT221845.1).

To analyse Y chromosome SNP, first, the amplification of X chromosome part was performed for all samples using fluorescently labeled probe unique complementary to the X chromosome (*PLP1* gene, ENSECAG00000015446) and the TaqMan™ Gene Expression Master Mix kit (Thermo Fisher Scientific). The X chromosome were used as an internal control. Next, all of the male samples were genotyped using the allelic discrimination method with assay complementary to the Y chromosome (designed using Primer Express™) and TaqPath™ ProAmp™ Master Mix (Thermo Fisher Scientific). The SNP localized on the Y chromosome and distinguishing *Equus caballus* from *Equus przewalskii* was investigated (MH341179.1, g731821T>C), [16]. Samples were analysed qualitatively, the probes with X and Y signals were recognized as male (Table 1). Real-time PCR reaction was performed on QuantStudio™ 7 Flex (Thermo Fisher Scientific).

**Table 1:** The primer sequence and detailed information about analysed polymorphisms in Przewalski horses

Region	Analysed fragment	Identification method	Primers/probes	Length (bp)
Mitochondrial DNA	Amplicon 1	Sanger sequencing	F: AACGTTTCCTCCCAAGGACT R: GTAGTTGGGAGGGTTGCTGA	397
	Amplicon 2	Sanger sequencing	F: ACCCCATCCAAGTCAAATCA R: ACCCCATCCAAGTCAAATCA	462
	Amplicon 3	Sanger sequencing	F: ACCTACCCGCGCAGTAAGCAA R: ACGGGGGAAGAAGGGTTGACA	306
Sex chromosomes	Y chromosome	Allelic discrimination	F: AGACCCGCCGGTGC R: CAATTCCCTGGAGCCTCTGTAG VIC: AGTCCTGGTGAATGAG FAM: TCCTGGCGAATGAG	64
	X chromosome	Real-Time PCR reaction	F: GTCAGGCCAAGGAGAGTAGCA R: GCACATCCTCCTCCACTTATGC NED: CCCAGTTCTTAGGTCACA	73
*TBX* gene^a^	chr8:18,227,267 + 1066G>T	Sanger sequencing	F: TAAGCCTCCAGACACCCAAG R: CAGCTCCCGTCTCCCTAGAT	240
	chr8:18,226,905A>G	Sanger sequencing	F: TTCCAGGAACCTGAGCAAAT R: ATAACCAGGCACCCCTTCTC	155
	chr8:18,227,267, in/del	Amplification length polymorphism	F: CAAGACGCAAGGCTCTTTCT R: CGTTTCTTTAAGGGCTCGTG	In 1.837 Del 215
*MC1R* gene	ECA3g.36,259,552C>T	Sanger sequencing	F: CCTACCTCGGGCTGACCACCAA R: GAGAGGACACTAACCACCCAGATG	276

^a^Stefaniuk-Szmukier et al., [17]

Moreover, two coat color genes were investigated: *TBX3* and *MC1R*. All PCRs were carried out with Phanta Ready Mix (Vazyme Biotech). Two polymorphisms at the *TBX3* gene (chr8:18,227,267 + 1066G>T, chr8:18,226,905A>G) were detected by Sanger sequencing and the heterozygosity state of deletion (1.6 kb in/del, chr8:18,227,267) was investigated by the amplification length polymorphism method using method described by Stefaniuk-Szmukier et al. [17]. Melanocortin-1-receptor (*MC1R*) gene polymorphism responsible for ‘fox’ color (ECA3g.36,259,552C>T) was detected by Sanger sequencing.

## Results and discussion

**Fig. 1:**
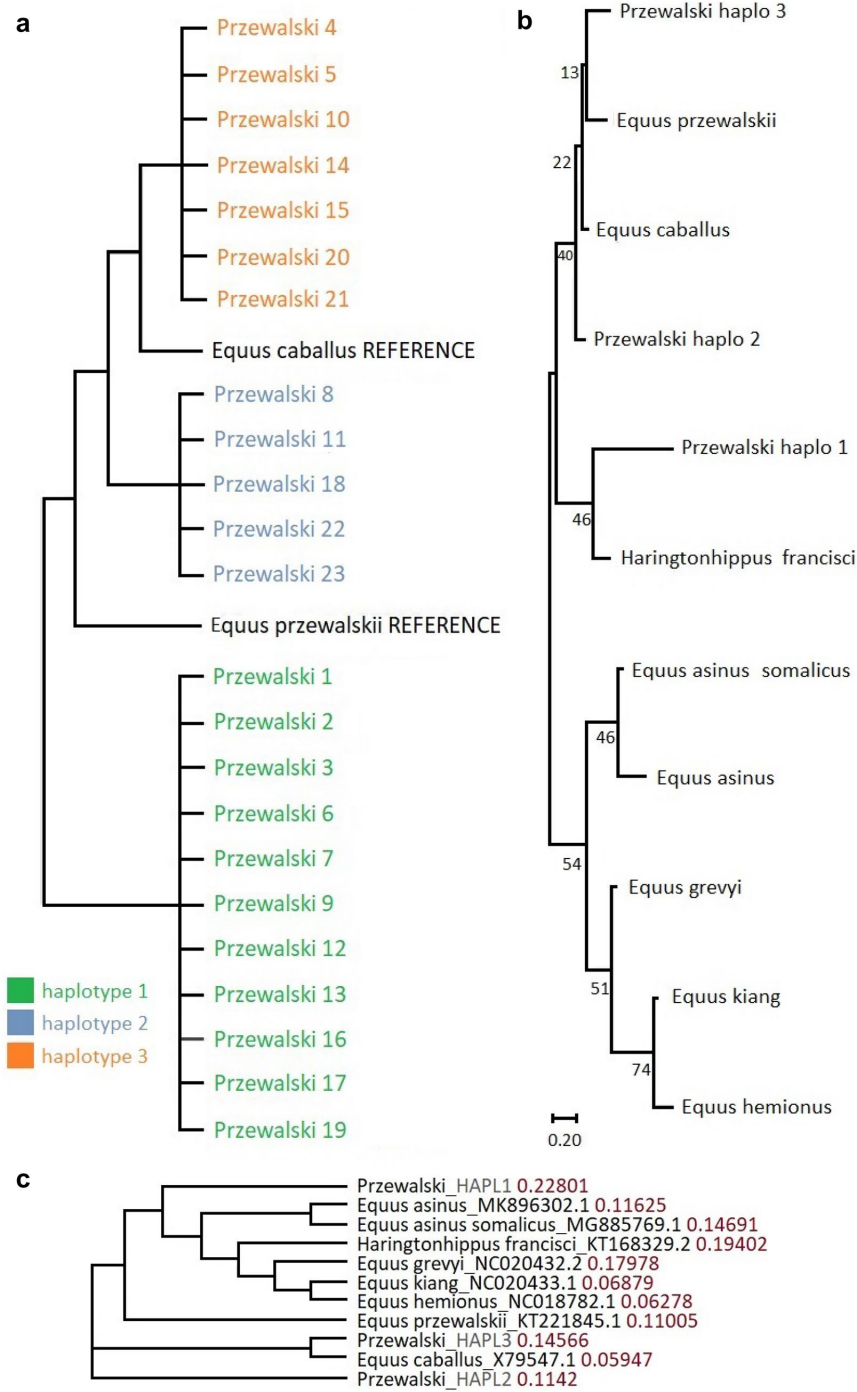
**a** The neighborhood tree illustrates mtDNA sequence similarities between 23 analysed Przewalski horses and the haplotypes which they are presenting (Mega 11.0.9 software).** b** The neighborhood tree illustrates mtDNA distances between three Przewalski horse haplotypes and the species selected as potentially having the highest mtDNA sequence similarity to them based on NCBI reference with the substitution parameter (Mega 11.0.9 software).** c** The neighborhood tree illustrates mtDNA distances between three Przewalski horse haplotypes and the species selected as potentially having the highest mtDNA sequence similarity to them (Mega 11.0.9 software)

The sequencing of mitochondrial DNA hypervariable region 1 and hypervariable region 2 assigned Przewalski horses to the three distinctly different haplotypes (Fig. 1a). All samples were clustered in three homogeneous groups presenting unique SNPs patterns (Table 2). The obtained sequences have been submitted to GenBank and received accession numbers: ON393914, ON393915, ON393916. The mtDNA haplotypes comparison with other Equidae mtDNA sequences available in GenBank was determined qualitatively using the evolutionary distance values (in Figs. 1b and c) and showed an interesting association. The haplotype 3 is uniquely clustered with *Equus caballus* reference, while the haplotype 2 is separated from the genus *Equus*, showing almost the same value of evolutionary distance with *Equus przewalskii* reference (KT221845.1; 0.1142 and 0.11005 respectively) and is probably low admixtured with other Equidae. The haplotype 1 presented the evolutionary distance value similarity to the *Haringtonhippus* (KT168329.2; 0.22801 and 0.19402 respectively)—extinct species, that lived in North America during the Pleistocene.

**Table 2:** The mitochondrial DNA haplotypes occurrence in different species

	15538	15542	15585	15595	15597	15650	15666	15709	15762	15766	15769	15770	15771	15775	15776
*Equus caballus* **(reference)**	A	C	G	A	A	A	G	C	G	C	T	C	C	C	T
Przewalski hap.1	G	C	G	A	G	G	G	T	A	T	C	T	C	T	G
Przewalski hap.2	A	C	A	A	A	A	G	C	G	C	T	C	T	T	T
Przewalski hap.3	A	T	G	G	A	G	A	C	G	C	T	C	C	C	T
*Equus przewalskii* (reference)	G	C	G	A	G	G	G	T	G	C	T	C	T	C	T

	15807	15811	15826	15827	15868	15870	15871	15956	15995	16007	16022	16055	16514	16557/58
*Equus caballus* **(reference)**	C	C	A	A	T	C	C	A	A	T	T	G	C	–
Przewalski hap.1	T	T	A	G	C	T	C	G	G	C	C	A	C	inC
Przewalski hap.2	C	C	A	A	T	T	T	A	A	T	T	A	C	inC
Przewalski hap.3	C	C	A	A	C	T	C	A	A	T	T	A	T	–
*Equus przewalskii* (reference)	C	C	G	A	T	C	C	A	A	T	T	A	C	–

*Haringtonhippus* was described by Heintzman et al. [18] as the result of the full mitochondrial and partial nuclear genomes analysis from late Pleistocene ‘New World stilt-legged’ equids endemic to North America. They also demonstrated that *Haringtonhippus* falls outside of a crown group *Equus* however, based on our results, it can be suspected that the *Haringtonhippus* may have interbred with selected equines, including Przewalski horses. The geographical barrier concerning both species occurrence could be overcome through the land existing in the Pleistocene and connecting today’s Siberia in Asia with North America—called Beringia. Therefore, the gene flow between *Haringtonhippus* and *Equus przewalskii* populations may have occurred regularly at least periodically during the Pleistocene. Moreover, the latest research confirms a small proportion of North American ancestry in Przewalski horses and observed that in present Przewalski horses some of their genomic origin is derived from relatively recent gene flow from extinct North American horses [19].

According to the Y chromosome analysis, 13 of 23 samples were male and all of them presented C allele in MH341179.1 g731821T>C *locus*, characteristic for Przewalski horses. The research previously conducted on Przewalski horses [16] also presented C allele in this location and confirmed the g731821T>C substitution characteristics only for Przewalski horses.

The coat color genes analyses indicated only native, wild genotypes. The *TBX3* gene polymorphisms were analysed. SNP1 (G in Dun, T in non-dun1) is located within the region deleted in non-dun2, 1067 bp downstream of the deletion breakpoint at chr8:18,227,267. SNP2 (G in Dun, A in non-dun1 and non-dun2), is located 362 bp upstream of the deleted region in non-dun2 at chr8:18,226,905. The results assigned all horses to the genotype with G/G alleles in both SNP *loci*, and deletions were not observed. *MC1R* variants were also identified; none of Przewalski horses presented C to T missense mutation associated with an unfavorable ‘fox’ color variant for this species. The ability to produce phaeomelanin is attributed to the recessive alleles ‘ee’ and is not in concordance with the recommendations for the Przewalski horses [12].

## Conclusion

The presented genetic analysis of Przewalski horses population living in the Askania-Nova reserve adds useful information for the conservation of the breed. The mtDNA results, according to the literature, confirm three pure and fixed remaining maternal lineages in Przewalski horses, assigning the individuals to three distinctly different haplotypes. The Y chromosome polymorphism analysis confirmed the presence of genotype C characteristics for Przewalski horses in all 13 male individuals, moreover, the polymorphisms within the coat color genes *MC1R* and *TBX3* indicated only native, wild genotypes. Unexpected polymorphisms’ appearance in genes determining the coat color and in Y chromosome could indicate population crossbreeding, therefore the homogeneity in these genes and the Y chromosome in the Askania-Nova population needed to be confirmed.

## Data Availability

The data will be provided on request by the corresponding author. The manuscript has been already submitted to a preprint platform Research Square and recieved DOI: 10.21203/rs.3.rs-1684262/v1; this work is under a CC BY 4.0 License.

## References

[1] Orlando L, Ginolhac A, Zhang G, Froese D, Albrechtsen A (2013). Recalibrating Equus evolution using the genome sequence of an early Middle Pleistocene horse. Nature.

[2] Lau AN, Peng L, Goto H, Chemnick L, Ryder OA, Makova KD (2009). Horse Domestication and Conservation Genetics of Przewalski’s horse inferred from sex chromosomal and autosomal sequences. Mol Biol Evol.

[3] Gaunitz C, Fages A, Hanghøj K, Albrechtsen A, Khan N (2018). Ancient genomes revisit the ancestry of domestic and Przewalski’s horses. Science.

[4] Librado P, Orlando L (2021). Genomics and the Evolutionary history of Equids. Annu Rev Anim Biosci.

[5] Foose TJ, Lacy RC, Princee F, Ryder O, Seal US, Zimmermann W (1990) Przewalski’s Horse Global Conservation Plan Draft, IUCN Species Survival Commission e-Books

[6] Wallner B, Brem G, Muller M, Achmann R (2003). Fixed nucleotide differences on the Y chromosome indicate clear divergence between Equus przewalskii and Equus caballus. Anim Genet.

[7] Kerekes V, Sándor I, Nagy D, Ozogány K, Göczi L, Ibler B, Széles L, Barta Z (2021). Trends in demography, genetics, and social structure of Przewalski’s horses in the Hortobagy National Park, Hungary over the last 22 years. Global Ecol Conserv.

[8] Zharkikh TL, Yasynetska NI (2007). Preservation of gene pool of the Przewalski horses at Askania Nova: breeding in lines.

[9] Zvegintsova NS, Zharkikh T, Kuzmina T (2019). Parasites of Przewalski’s horses (Equus ferus przewalskii) in Askania Nova Biosphere Reserve (Ukraine) and Orenburg State Nature Reserve (Russia). Nat Conserv Res.

[10] Goto H, Ryder OA, Fisher AR, Schultz B, Kosakovsky Pond SL, Nekrutenko A, Makova KD (2011). A massively parallel sequencing approach uncovers ancient origins and high genetic variability of endangered Przewalski’s horses. Genome Biol Evol.

[11] Kvist L, Niskanen M (2021). Modern northern domestic horses carry mitochondrial DNA similar to Przewalski’s horse. J Mammal Evol.

[12] Thiruvenkadan AK, Kandasamy N, Panneerselvam S (2008). Coat colour inheritance in horses. Livest Sci.

[13] Ludwig A, Pruvost M, Reissmann M, Benecke N, Brockmann GA, Castaños P, Cieslak M, Lippold S, Llorente L, Malaspinas AS, Slatkin M, Hofreiter M (2009). Coat color variation at the beginning of horse domestication. Science.

[14] Reissmann M, Musa L, Zakizadeh S, Ludwig A (2016). Distribution of coat-color-associated alleles in the domestic horse population and Przewalski’s horse. J Appl Genet.

[15] Imsland F, McGowan K, Rubin C-J, Henegar C, Sundström E, Berglund J (2015). Regulatory mutations in *TBX3* disrupt asymmetric hair pigmentation that underlies dun camouflage color in horses. Nat Genet.

[16] Wallner B, Vogl C, Shukla P, Burgstaller JP, Druml T, Brem G (2013). Identification of genetic variation on the horse Y chromosome and the tracing of male founder lineages in modern breeds. PLoS ONE.

[17] Stefaniuk-Szmukier M, Ropka-Molik K, Piórkowska K, Szmatoła T, Długosz B, Pisarczyk W, Bugno-Poniewierska M (2017). Variation in *TBX3* gene region in dun coat color Polish Konik horses. J Equine Veterinary Sci.

[18] Heintzman PD, Zazula GD, MacPhee RDE, Scott E (2017). A new genus of horse from Pleistocene North America. eLife.

[19] Vershinina AO, Heintzman PD, Froese DG, Zazula G, Cassatt-Johnstone M (2021). Ancient horse genomes reveal the timing and extent of dispersals across the Bering Land Bridge. Mol Ecol.

